# The Control of Diastolic Calcium in the Heart: Basic Mechanisms and Functional Implications

**DOI:** 10.1161/CIRCRESAHA.119.315891

**Published:** 2020-01-30

**Authors:** David A. Eisner, Jessica L. Caldwell, Andrew W. Trafford, David C. Hutchings

**Affiliations:** From the Unit of Cardiac Physiology, Division of Cardiovascular Sciences, University of Manchester, United Kingdom.

**Keywords:** calcium, diastole, heart failure, myofibrils, stroke volume

## Abstract

Normal cardiac function requires that intracellular Ca^2+^ concentration be reduced to low levels in diastole so that the ventricle can relax and refill with blood. Heart failure is often associated with impaired cardiac relaxation. Little, however, is known about how diastolic intracellular Ca^2+^ concentration is regulated. This article first discusses the reasons for this ignorance before reviewing the basic mechanisms that control diastolic intracellular Ca^2+^ concentration. It then considers how the control of systolic and diastolic intracellular Ca^2+^ concentration is intimately connected. Finally, it discusses the changes that occur in heart failure and how these may result in heart failure with preserved versus reduced ejection fraction.

So then I could tell them

Where the wind goes…

But where the wind comes from

Nobody knows.

—AA Milne, “Wind on the Hill”

In keeping with the above quotation from the collection of poems for children by A.A. Milne,^1^ the focus of this article is not on the extensively studied mechanisms that deliver calcium ions to the myofilaments and thereby produce systole. Rather, we review the much less well understood removal of Ca^2+^. Specifically, we will consider how diastolic intracellular Ca^2+^ concentration ([Ca^2+^]_i_) is controlled and how it changes in disease.

## Why Is It Important to Control Diastolic [Ca^2+^]_i_?

### Mechanical Relaxation

An upper limit for diastolic [Ca^2+^]_i_ results from the need for the myofilaments to be deactivated to allow ventricular filling. There may, however, be reasons for ensuring that diastolic [Ca^2+^]_i_ is not too low as, the lower it is, to reach a given systolic level, more Ca^2+^ must be added to and removed from the cytoplasm on each beat. This will increase energy expenditure, and since Ca^2+^ cycling accounts for about 30% of the energy consumption of the myocardium,^2^ this may be a significant factor in requiring that diastolic [Ca^2+^]_i_ is not too low.

### Diastolic Influences Systolic [Ca2+]i and Force

There are 2 factors. (1) The lower the diastolic [Ca^2+^]_i_, the more Ca^2+^ must be added to produce a given increase in [Ca^2+^]_i_. This is because, at low [Ca^2+^]_i_, the cytoplasmic Ca^2+^ buffers become less saturated and their ability to absorb Ca^2+^ increases. Conversely, as [Ca^2+^]_i_ increases, buffering power will decrease so a given increase in [Ca^2+^]_i_ will require a smaller increase in total Ca^2+^ (for review, see the article by Smith and Eisner^3^). In other words, by altering the level of saturation of buffers, diastolic [Ca^2+^]_i_ determines the amplitude of the systolic transient produced by a given rise of total Ca^2+^ and therefore alterations of diastolic [Ca^2+^]_i_ change the inotropic response. (2) A further consideration is that force depends steeply on [Ca^2+^]_i_ so that, starting from an elevated diastolic [Ca^2+^]_i_, a smaller increase in [Ca^2+^]_i_ will be required to produce the same change of force compared with at a normal diastolic [Ca^2+^]_i_.^4^ Therefore, an increase in diastolic [Ca^2+^]_i_ will increase the level of developed force produced by a given systolic rise of [Ca^2+^]_i_.

## Basic Mechanisms Underlying the Ca Transient

**Figure 1.**
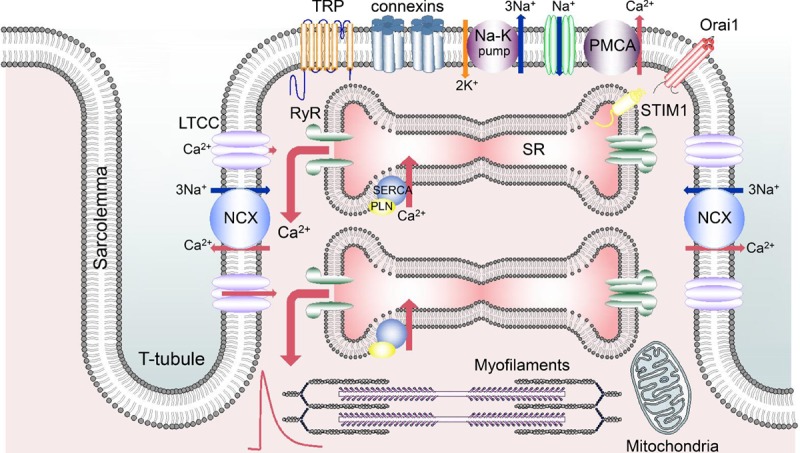
**Pathways involved in cardiac cellular calcium cycling.** The schematic shows part of a ventricular myocyte including transverse (T) tubules. From left to right, the sarcolemma contains sodium-calcium exchange (NCX); L-type Ca channel (LTCC); transient receptor potential (TRP) channels; connexin hemichannels; Na-K pump; Na channel; PMCA (plasma membrane Ca-ATPase); Orai. The sarcoplasmic reticulum (SR) contains ryanodine receptor (RyR); SR Ca-ATPase (SERCA) and its regulatory protein, PLN (phospholamban); STIM1 (stromal interaction molecule 1).

The pathways that underlie cardiac calcium cycling are well understood^5,6^ (Figure 1); the individual mechanisms and their roles in the control of diastolic [Ca^2+^]_i_ are described in more detail in subsequent sections. Briefly, Ca^2+^ enters via the L-type Ca channel, and there may also be entry on reverse sodium-calcium exchange (NCX) at the start of the action potential. This Ca^2+^ entry triggers the release of a larger amount of Ca^2+^ from the sarcoplasmic reticulum (SR) through the ryanodine receptor (RyR)—a process known as calcium-induced calcium release. Ca^2+^ is then returned to the SR by the SR Ca-ATPase (SERCA), regulated by the accessory protein PLN (phospholamban). At the surface membrane, Ca^2+^ is removed from the cell by a combination of NCX and PMCA (plasma membrane Ca-ATPase). Finally, the mitochondria can uptake Ca^2+^ via the MCU (mitochondrial calcium uniporter). The amplitude of the systolic rise of [Ca^2+^]_i_ is increased by increasing the size of the L-type Ca current^7,8^ or the amount of Ca stored in the SR.^9,10^ The latter is determined by the balance of cellular Ca^2+^ fluxes. For example, increasing SERCA activity or decreasing Ca efflux on NCX will increase SR Ca^2+^ content. The decay of the Ca transient is largely due to SERCA-mediated reuptake into the SR with, particularly in larger species, significant contributions from NCX.^4^ The rate of this decay would be expected to affect end-diastolic [Ca^2+^]_i_ since, all other things being equal, a faster decay will mean that [Ca^2+^]_i_ is reduced to a lower level by the time of the next beat, resulting in a lower end-diastolic [Ca^2+^]_i_.

## Why Is So Little Known About Diastolic [Ca^2+^]_i_?

**Figure 2.**
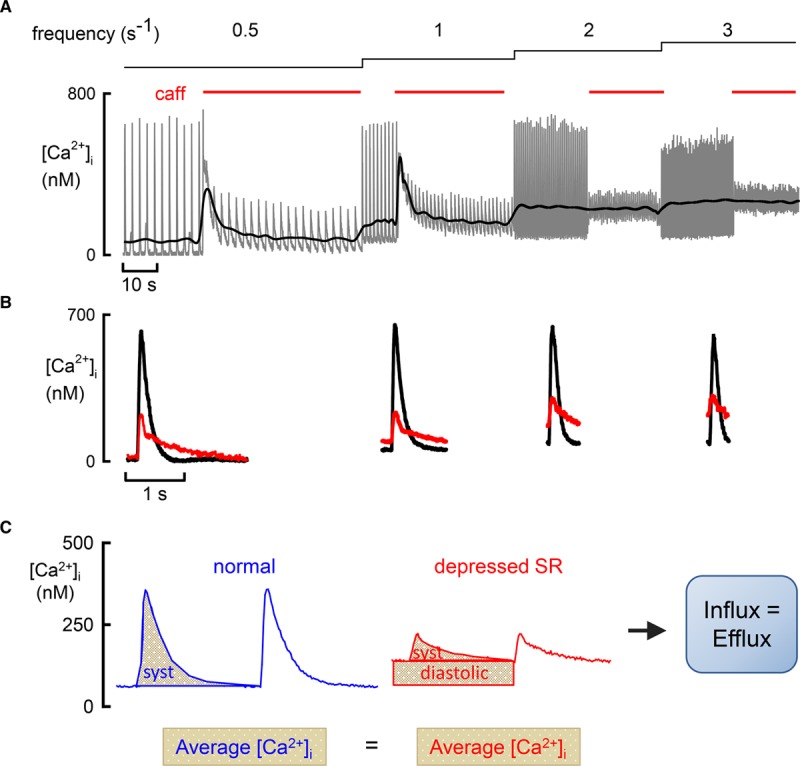
**The importance of average intracellular Ca2+ concentration ([Ca2+]i) in the control of systolic (syst) and diastolic [Ca2+]i.** **A**, The effects of application of caffeine (caff) and stimulation rate on [Ca^2+^]_i_. A rat ventricular myocyte was stimulated at the frequencies shown above and caff (1 mmol/L) applied for the periods denoted by the red bars. The gray trace is the original data and the black denotes average [Ca^2+^]_i_. **B**, Specimen, averaged traces of [Ca^2+^]_i_ from the frequencies shown above. For each frequency, the control (black) and caff (red) traces are superimposed. Data reproduced from Sankaranarayanan et al.^14^ **C**, Illustration of flux balance in control and with depressed sarcoplasmic reticulum (SR) function. The total Ca^2+^ efflux via sodium-calcium exchange (NCX) above control diastolic levels is represented by the area under the [Ca^2+^]_i_ trace. In the depressed SR case, this is separated into 2 components: (1) activated by the syst Ca^2+^ transient and (2) activated by increased diastolic [Ca^2+^]_i_. Average [Ca^2+^]_i_ is identical with normal and depressed SR (**A**), and, therefore, Ca^2+^ efflux is unchanged and equal to influx.

There are several reasons for the paucity of data concerning diastolic [Ca^2+^]_i_. (1) Problems of indicator calibration make it much easier to measure changes than absolute levels of [Ca^2+^]_i_. This is a particular issue when comparing measurements between cells or animals. (2) When nonratiometric, Ca^2+^-sensitive, fluorescent indicators are used, the records are often normalized to the diastolic or resting fluorescence,^11^ making it difficult to measure diastolic [Ca^2+^]_i_. (3) In experiments using the whole cell version of the patch clamp, diffusion of Ca^2+^ and Ca^2+^ buffers into or out of the pipette may contribute to regulation of [Ca^2+^]_i_. Indeed, one of the uses of the whole-cell technique is to control the cytoplasmic ionic concentrations. (4) The major issue may be that, particularly in smaller animals, most experimental work studying Ca^2+^ cycling in cardiac tissues has used rates of stimulation considerably below normal heart rates. While the fact that ion currents and [Ca^2+^]_i_ have reached steady state values helps dissect the fluxes responsible for the systolic Ca transient, it establishes an artificial situation. As discussed below, end-diastolic [Ca^2+^]_i_ represents a balance between many Ca^2+^-handling mechanisms. In contrast, in a quiescent myocyte, the resting level of [Ca^2+^]_i_ is determined entirely by the fluxes of Ca^2+^ across the sarcolemma^12,13^ because, in the steady state, there can be no net flux into or out of organelles. Such a net flux would result in a continuous change of organelle Ca^2+^ content—a situation incompatible with a steady state. At low rates of stimulation, [Ca^2+^]_i_ will be identical to the resting level seen in the unstimulated case. These frequency-dependent effects are illustrated by making the RyR leaky with caffeine (Figure 2A and 2B). This has no effect on diastolic [Ca^2+^]_i_ at a stimulation rate of 0.5 Hz but a marked one at 3 Hz.^14^ Thus, it is important not to confuse diastolic and resting [Ca^2+^]_i_. Finally, as discussed below, physiological changes of heart rate result from those of autonomic tone—a factor that is not examined in studies that simply alter pacing rate.

## Interaction of Control of Diastolic and Systolic [Ca^2+^]_i_

It is tempting to think of the control of diastolic and systolic [Ca^2+^]_i_ as being separate. From this viewpoint, diastolic [Ca^2+^]_i_ is controlled at a certain level, and the mechanisms discussed above determine the magnitude of the systolic rise. We think that this is incorrect; the regulation of diastolic and systolic [Ca^2+^]_i_ is inextricably linked.^14,15^ This has been demonstrated recently by investigating the effects of interfering with SR function on diastolic and systolic [Ca^2+^]_i_. Consistent with previous data,^16^ making the RyR leaky with caffeine decreased the amplitude of the systolic Ca^2+^ transient by decreasing SR Ca^2+^ content. This was accompanied by an increase in diastolic [Ca^2+^]_i_ such that the average level of [Ca^2+^]_i_ over the cycle was unaffected (Figure 2A).^14^ Similar results were found when SERCA activity was decreased with thapsigargin and were accounted for by considerations of cellular Ca^2+^ flux balance. In the steady state, the Ca^2+^ influx over the cardiac cycle must equal efflux. Interfering with SR function will have no direct effect on influx, and so efflux must also be unaltered. Since Ca^2+^ efflux on NCX is proportional to [Ca^2+^]_i_,^17^ constant efflux requires that average [Ca^2+^]_i_ be unaffected, explaining why the decrease of systolic is accompanied by an increase in diastolic [Ca^2+^]_i_ (Figure 2C). One caveat is required here; interfering with SR function and thereby decreasing the amplitude of the systolic Ca transient can decrease the degree of Ca-dependent inactivation of the L-type Ca current and thereby increase Ca^2+^ influx.^18,19^ In this case, average [Ca^2+^]_i_ would be elevated, potentially elevating diastolic [Ca^2+^]_i_. This does not appear to be an issue in experiments where the RyR was made leaky with caffeine as the L-type Ca^2+^ influx was unaffected.^14^

### Importance of Average [Ca^2+^]_i_

**Figure 3.**
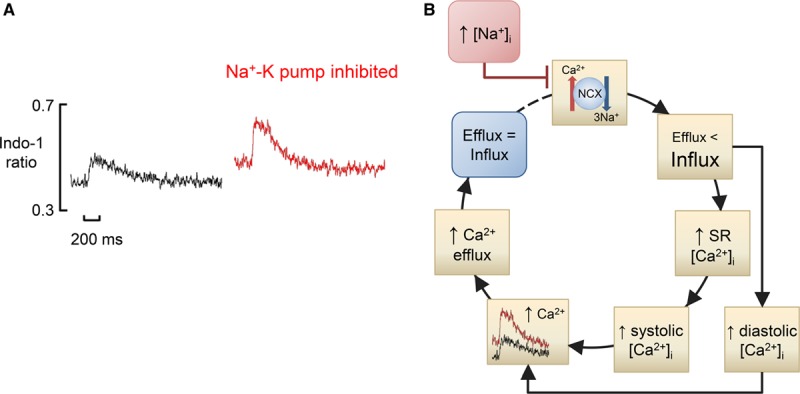
**Mechanism of the effects of intracellular Na+ concentration ([Na+]i) on diastolic intracellular Ca2+ concentration ([Ca2+]i).** **A**, Original data. Ca transients recorded using indo-1 in a rat ventricular myocyte. Records show the following: left, control; right, in the presence of the Na-K pump inhibitor, strophanthidin. Data reproduced from Bennett et al.^20^ **B**, Flowchart. Initially (blue rectangle), Ca^2+^ efflux equals influx. The increase in [Na^+^]_i_ decreases sodium-calcium exchange (NCX) activity making Ca^2+^ efflux less than influx, leading to an increase in diastolic [Ca^2+^]_i_. There will also be an increase in sarcoplasmic reticulum (SR) Ca^2+^ content and thence an increase in systolic [Ca^2+^]_i_. The increases of diastolic and systolic [Ca^2+^]_i_ will increase Ca^2+^ efflux until efflux again equals influx.

The average [Ca^2+^]_i_ is determined by Ca^2+^ entry and efflux across the surface membrane. An increase in rate will increase Ca^2+^ influx per unit time and thence the average [Ca^2+^]_i_ (Figure 2A).^14^ Similar effects would be expected for an increase in the amplitude of the L-type Ca current. Conversely, a decrease in the ability of NCX to pump Ca^2+^ out of the cell will increase average [Ca^2+^]_i_ to a level sufficient to maintain Ca^2+^ efflux. This may arise due to either decreased expression of NCX or an increase in the intracellular Na^+^ concentration ([Na^+^]_i_) and, therefore, a decrease in the energy to pump Ca^2+^ out of the cell (Figure 3). There is an infinite number of combinations of systolic and diastolic [Ca^2+^]_i_ that can establish a given average [Ca^2+^]_i_. The properties of the SR will be an important factor in determining which occurs. Compromising SR function, by decreasing SERCA activity or increasing Ca^2+^ (leak) efflux through the RyR, will increase diastolic and decrease systolic [Ca^2+^]_i_. For example, in the presence of a normal SR, β-adrenergic stimulation increases systolic but has no effect on diastolic [Ca^2+^]_i_. In contrast, when the RyR is leaky, β-stimulation increases diastolic [Ca^2+^]_i_.^14^

Similar considerations also apply to conditions of calcium overload where waves of calcium release from the SR occur. Much attention has been directed to the detrimental effects of these increases in [Ca^2+^]_i_ which activate NCX^21^ and thereby produce arrhythmogenic delayed afterdepolarizations.^22^ However, the resulting Ca^2+^ efflux will help maintain Ca^2+^ flux balance and thereby keep diastolic [Ca^2+^]_i_ lower than would otherwise be the case. Under these conditions, addition of caffeine to empty the SR and thereby remove waves and their associated efflux results in a steady level of [Ca^2+^]_i_, which is greater than the minimum seen in the presence of Ca^2+^ waves.^23^

These consequences of flux balance are a generalization of those previously described for changes of systolic [Ca^2+^]_i_ alone.^24–26^ That work showed that potentiation of RyR opening had no effect on systolic [Ca^2+^]_i_ in the steady state. In those earlier experiments, the decrease of SR Ca content was exactly compensated for by an increase in fractional release from the SR so the amplitude of the Ca transient and the accompanying Ca^2+^ efflux were unaltered. Ca^2+^ flux balance could, therefore, be maintained at constant diastolic [Ca^2+^]_i_. In the more recent work,^14^ the degree of potentiation of the RyR was greater (higher concentrations of caffeine used), and, therefore, the SR Ca content fell to such a low level that even if it is all released, the systolic Ca transient is smaller than control. The consequent decrease of systolic Ca^2+^ efflux results in systolic efflux being less than influx, thereby loading the cell with Ca^2+^ and increasing diastolic [Ca^2+^]_i_ until the increase of diastolic efflux returns the cell to Ca^2+^ flux balance.

In the context of the above general considerations, we will review important aspects of the underlying Ca^2+^ fluxes before discussing how their integration leads to control of diastolic and systolic [Ca^2+^]_i_.

## Fluxes Regulating Diastolic [Ca^2+^]_i_

### Ca^2+^ Buffering

The changes of [Ca^2+^]_i_ potentially depend as much on the Ca^2+^ buffering properties of the cell as on the fluxes of total Ca^2+^.^3^ In quiescent cells (or at low pacing rates), an increase in buffering is not expected to change diastolic [Ca^2+^]_i_, since free [Ca^2+^]_i_ (and not the Ca^2+^ bound to buffers) determines efflux, and this must balance influx, which is constant. At higher pacing rates, because an increase in buffering slows the rate of change of [Ca^2+^]_i_, the Ca transient cannot decay back to baseline and end-diastolic [Ca^2+^]_i_ will rise. Accordingly, experimentally increasing the cytoplasmic buffering power slows the rate of decay of [Ca^2+^]_i_^27^ and elevates [Ca^2+^]_i_ and force in diastole.^28^ An increase in diastolic [Ca^2+^]_i_ in hypertrophic cardiomyopathy resulting from some troponin T mutations has been attributed to this mechanism^29^ and may contribute to contractile impairment at increased heart rates in this condition.

### Sarcoplasmic Reticulum Ca-ATPase

The greater the activity of SERCA, the faster systolic [Ca^2+^]_i_ will decay, and, all other things being equal, the further diastolic [Ca^2+^]_i_ will fall before the next beat and, therefore, the lower will be end-diastolic [Ca^2+^]_i_. Experimentally decreasing SERCA activity can (see above) increase diastolic [Ca^2+^]_i_^14,16,30^ and pressure^30^ as a consequence of the slowing of the decay of the Ca^2+^ transient. The increased diastolic [Ca^2+^]_i_ will compensate for the decreased systolic efflux resulting from the smaller Ca^2+^ transient thereby maintaining Ca^2+^ flux balance. It should, however, be noted that acute inhibition of SERCA has been reported to increase [Na^+^]_i_,^31^ and this can elevate diastolic [Ca^2+^]_i_ via NCX. The origin of this increase in [Na^+^]_i_ is unclear. One possibility is that the decreased amplitude of the Ca^2+^ transient will have decreased inactivation of the L-type Ca current, thereby increasing Ca^2+^ entry and thence efflux on NCX, leading to loading of the cell with Na^+^. Given that Na^+^ entry on NCX is a major component of Na+ entry into the cell,^32^ this will elevate [Na^+^]_i_. In another study, knockout of SERCA also elevated [Na^+^]_i_.^33^ These knockout mice have increased L-type Ca current, possibly to compensate for the lack of SERCA. This increased Ca^2+^ influx will need to be balanced by increased efflux on NCX. The consequent increase in Na^+^ influx may, therefore, account for the elevation of [Na^+^]_i_.

### Ryanodine Receptor

As mentioned in a previous section, making the RyR leaky can decrease SR Ca content and thence the amplitude of the systolic Ca transient and systolic Ca^2+^ efflux. The decrease of efflux means that Ca^2+^ will accumulate in the cell, increasing diastolic [Ca^2+^]_i_ until the increase in diastolic Ca^2+^ efflux restores total efflux to equal influx. Leaky RyRs also slow the rate constant of decay of the systolic Ca transient.^34,35^ Under normal conditions, Ca release from the SR occurs more or less synchronously, a few milliseconds after the start of depolarization, in response to the rise of [Ca^2+^]_i_ produced by the L-type current. Release from clusters of RyRs can be seen as calcium sparks.^11^ In contrast, after myocardial infarction, Ca sparks are observed on the falling phase of the systolic Ca transient.^15^ Increasing RyR phosphorylation and opening by overexpression of CaMKII (Ca^2+^/calmodulin-dependent protein kinase II)-δ_C_ also leads to the appearance of delayed calcium sparks, which will interfere with the decay of [Ca^2+^]_i_ and relaxation.^35^ Such late sparks have also been suggested to be a more general phenomenon particularly when the initial release of Ca^2+^ from the SR is depressed.^36^ A study in hypothyroid mice has linked the appearance of late sparks to impaired systolic and diastolic function.^37^

### Sodium-Calcium Exchange

NCX uses the energy provided by 3 Na^+^ entering to pump 1 Ca^2+^ out of the cell. This stoichiometry generates an electric current,^21,38^ and NCX activity is sensitive not only to the Na^+^ and Ca^2+^ concentration gradients but also to membrane potential; hyperpolarization increases and depolarization decreases net Ca^2+^ efflux. Depending on the ionic gradients and membrane potential, NCX can reverse direction with net Ca^2+^ influx coupled to Na^+^ efflux (reverse mode). At a normal resting potential, NCX works in the forward direction with Ca^2+^ efflux roughly proportional to [Ca^2+^]_i_.^17^ It should, however, also be noted that NCX is allosterically regulated by [Ca^2+^]_i_, thus limiting Ca^2+^ efflux at low [Ca^2+^]_i_.^39^ For an extensive review of NCX, see the article by Blaustein and Lederer.^40^

### Intracellular Sodium

An increase in [Na^+^]_i_ decreases the driving force available for NCX to remove Ca^2+^ from the cell and thereby increases developed force and the underlying systolic Ca transient. In rabbit ventricular myocytes, inhibition of the Na-K pump increases both [Na^+^]_i_ and diastolic [Ca^2+^]_i_.^41^ However, at least with moderate increases in [Na^+^]_i_, there is often no increase in diastolic [Ca^2+^]_i_^42,43^ or developed force/cell length.^44–46^ While this may result partly from the low sensitivity of force and some Ca^2+^ indicators to [Ca^2+^]_i_, it may also be explained as follows (Figure 3). In the steady state, the reduction of NCX activity will require an increase in average [Ca^2+^]_i_ (see above). At first, this will be largely provided by an increase in systolic [Ca^2+^]_i_ as a result of the increase in SR Ca content. Only with further reduction of NCX, perhaps because there is a limit to how much SR Ca content and thence systolic [Ca^2+^]_i_ can increase, will diastolic [Ca^2+^]_i_ increase appreciably.

### Plasma Membrane Ca-ATPase

In addition to NCX, the myocyte also expresses a PMCA whose contribution to Ca^2+^ efflux is less well established.^47^ It has been argued that the PMCA is irrelevant to the control of bulk cytoplasmic [Ca^2+^]_i_ and, instead, has a signaling function by controlling [Ca^2+^]_i_ in microdomains near caveolae.^48^ Work from the Bers Laboratory finds that the contribution of the PMCA to Ca^2+^ removal in a variety of species is typically <10% of that of NCX.^49^ We find a larger contribution; inhibiting NCX with Ni^2+^ leaves 25% to 33% of the Ca^2+^ removal from the cell functional in rat.^16,50^ A concern with the use of Ni^2+^ is that it may not completely inhibit NCX, but similar results are seen when NCX is stopped by removal of Na^+^ ions.^51,52^ The NCX-independent Ca^2+^ efflux is abolished by the nonspecific PMCA inhibitor carboxyeosin.^53,54^ A substantial role for PMCA is also suggested by work on myocytes isolated from NCX knockout mice. These animals live normally, and their ventricular myocytes have normal Ca^2+^ transients. There is no change of PMCA expression, and the myocytes maintain Ca^2+^ flux balance by decreasing Ca influx through the L-type Ca current to 20%—a level at which PMCA alone can presumably balance it.^55,56^ This suggests that PMCA makes a contribution equivalent to 25% of that of NCX in the wild type. One caveat is that, as in other studies, the rate of Ca^2+^ removal from the cell was assessed from the rate of fall of the caffeine-evoked rise of [Ca^2+^]_i_. The available data do not provide caffeine exposures of sufficient duration to obtain accurate measurements,^56^ and further work is required to establish the role of PMCA in the regulation of diastolic [Ca^2+^]_i_.

### Mitochondrial Ca^2+^ Handling

In principle, Ca^2+^ uptake and release from mitochondria could affect diastolic [Ca^2+^]_i_. As we have recently reviewed,^6^ there are conflicting reports in the literature with only some studies finding evidence in favor of beat-to-beat movements of Ca^2+^ into and out of mitochondria. On balance, at least in adult ventricular myocytes, while changes of mitochondrial [Ca^2+^] can be observed at slow rates of stimulation,^57^ they disappear at higher rates questioning their importance in regulating diastolic [Ca^2+^]_i_.^57^

### Ca^2+^ Influx Pathways During the Action Potential

The major route for Ca^2+^ entry during the action potential is the L-type Ca current.^58^ In some regions of the heart, particularly in nodal tissues, there are also contributions from the T-type Ca channel.^59^ The stoichiometry of NCX means that it can also contribute to Ca^2+^ influx during depolarization, but, under normal conditions, this is much smaller than that through the L-type channel.^60^ In heart failure, the increase in [Na^+^]_i_ will increase influx through NCX,^61^ and it is possible that the magnitude of Ca^2+^ influx through NCX may have been underestimated due to making measurements at slow rates where [Na^+^]_i_ is decreased.

Many studies have investigated the effects on systolic [Ca^2+^]_i_ of maneuvers that alter the L-type Ca current. Inspection of most data shows little effect on diastolic levels,^62,63^ but the majority of experiments were performed at slow rates or used the whole-cell patch clamp technique. We found that decreasing the L-type Ca current with cadmium in cells where diastolic [Ca^2+^]_i_ was elevated reduced diastolic [Ca^2+^]_i_.^14^ From first principles, one would expect 2 opposing effects.^64^ (1) Increased L-type Ca current will increase Ca^2+^ influx per unit time thereby requiring an increased average [Ca^2+^]_i_ to balance it. Depending on the conditions, this may be achieved by increased systolic or diastolic [Ca^2+^]_i_. (2) The increase in L-type current will increase Ca^2+^ release from the SR, increasing systolic Ca, thereby contributing to the elevated average without the need to increase diastolic. This latter effect, however, is limited as it is impossible to release >100% of SR content. It should also be noted that, at least under some conditions, increasing L-type Ca current does not increase SR content.^64^ Additionally, the increase in systolic [Ca^2+^]_i_ will increase the time taken for [Ca^2+^]_i_ to decay back to baseline, increasing the tendency for diastolic [Ca^2+^]_i_ to rise at shorter pacing intervals (higher heart rates). Quantitative considerations will determine whether the increase in systolic efflux is sufficient to balance the increase in influx or, alternatively, whether elevated diastolic [Ca^2+^]_i_ occurs.

### Background Ca^2+^ Entry Mechanisms

In the absence of stimulation, resting [Ca^2+^]_i_ is of the order of 100 nmol/L indicating that some kind of background Ca^2+^ entry pathway must exist to balance Ca^2+^ efflux on NCX. Such a pathway accounts for the fact that, even in a quiescent cell, after being emptied with caffeine, the SR can be refilled by a mechanism that requires extracellular Ca^2+^.^65^ As mentioned above, Ca^2+^ waves can occur in cells held at a fixed membrane potential,^23^ again indicating an influx pathway to balance efflux on NCX during the waves. The magnitude of this influx is roughly proportional to external Ca^2+^ concentration in the range ≤5 mmol/L.^23^ Subsequent work, examining the effects on resting [Ca^2+^]_i_ of abruptly removing external Ca^2+^, provided an estimate for the background Ca^2+^ influx of the order of 2 to 6 µmol/L per s in rat ventricular myocytes.^66^ A recent study estimated Ca^2+^ influx from measurements of average [Ca^2+^]_i_ (see above) and found a value of about 4 µmol/L per s.^14^ These values compare to an entry on each action potential via the L-type Ca current of the order of 5 to 10 µmol/L. Therefore, at normal heart rates (in a rat) of 5 s^−1^, the background influx will be of the order of 10% of that carried by the L-type current. As regards the mechanism of this influx, one study identified a Ca^2+^ entry mechanism that increased on hyperpolarization of the surface membrane and was blocked by the relatively nonspecific agent gadolinium (Gd^3+^).^67^ It is, therefore, important to consider the identity of this flux.

#### Connexin Hemichannels

One Ca^2+^ flux inhibited by Gd^3+^ is that carried by connexins.^68^ The majority of connexins are found as pairs, made up of 2 hemichannels, one in each of the 2 cell membranes at the intercalated discs. These allow current to flow between cells. However, some connexins are present as hemichannels in the surface membrane of a single cell^69,70^ and may, therefore, provide a route for Ca^2+^ entry. Recent work has suggested that this entry may be increased in experimental cardiomyopathy induced by plakophilin-2 deficiency.^71^

#### Transient Receptor Potential Channels

Transient receptor potential (TRP) channels are also sensitive to Gd^3+^, and considerable work has investigated their role in the heart. Knockout of TRPV2 decreases the amplitude of the systolic Ca transient and contraction.^72^ The compound probenecid, which activates TRPV2, was also shown to increase contractility,^73^ and a small trial has shown that this compound improves cardiac function in patients with heart failure.^74^ It should, however, be noted that probenecid has other actions including inhibiting organic anion transporters.^75^ Furthermore, inhibition of TRPV4 decreases SR Ca release.^76^ The cardiomyopathy found in the mdx mouse model of muscular dystrophy is associated with elevated diastolic [Ca^2+^]_i_, which can be blocked by Gd^3+^, and has been attributed to Ca^2+^ entry via TRPC channels.^77^ Similar results were found for the experimental myopathy produced by infusion of isoproterenol.^78^ Further evidence suggesting a role for TRP channels in contributing to setting diastolic [Ca^2+^]_i_ comes from the observation that knocking out both TRPC1 and TRPC4 in mice decreased diastolic [Ca^2+^]_i_.^79^ The recent synthesis of specific antagonists of TRPC channels^80^ and an agonist^81^ should make it possible to study the role of these channels more precisely.

TRP channels have also been implicated in the influx of Ca^2+^ into the cell activated by emptying the SR, so called store-operated Ca^2+^ entry, and in the HL-1 cell line, this has been suggested to contribute to resting [Ca^2+^]_i_.^82^ One issue is that much of the evidence for a role of store-operated channels in cardiac tissue comes from work on cultured or neonatal cells,^83,84^ and these may not be representative of adult myocytes. Some recent articles have, however, reported store-operated Ca^2+^ entry into adult mouse ventricular myocytes^85^ with the fluxes being inhibited by Gd^3+^.^86,87^ In many tissues, store-operated calcium entry is produced by a combination of the endoplasmic reticulum Ca^2+^ sensor STIM1 (stromal interaction molecule 1) and the surface membrane channel Orai1 (see the article by Qiu and Lewis^88^ for review). Overexpression of STIM1 in mouse heart increases diastolic [Ca^2+^]_i_, as a result of increased Ca^2+^ entry into the cell and increased leak from the SR.^89^ It should, however, be noted that STIM1 has also been reported to interact with PLN and thereby control SERCA.^90^

Finally, some TRP channels and connexins transport Na^+^ in addition to Ca^2+^ and, by altering [Na^+^]_i_, could affect [Ca^2+^]_i_ indirectly via NCX. All in all, it is clear that more work is required to characterize the contribution of TRPs, connexins, and as yet unidentified mechanisms to the background Ca^2+^ influx

## Physiological Factors Affecting Diastolic [Ca^2+^]_i_

### Heart Rate

Increasing the rate of stimulation increases diastolic [Ca^2+^]_i_ in ventricular trabeculae^91^ and isolated myocytes.^14,92–95^ It is important to note that the Ca^2+^ indicators used to measure [Ca^2+^]_i_ buffer [Ca^2+^]_i_ to some degree and potentially exaggerate the effects of increased frequency. It would be useful to repeat these experiments using as low concentrations of Ca indicators as possible. Of course the fact that, even in the absence of indicators, increasing stimulation rate increases diastolic force^96^ and decreases cell length^92^ means that excessive buffering cannot account for all the effects.

**Figure 4.**
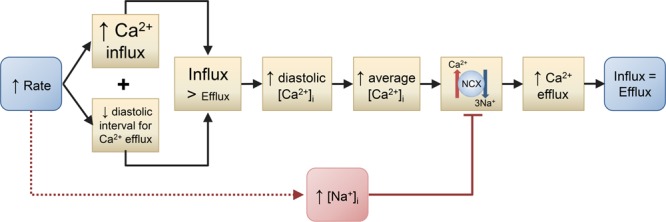
**Possible mechanisms for the increase in diastolic intracellular Ca2+ concentration ([Ca2+]i) with increased stimulation rate.** Two mechanisms are shown. (1) The increase in rate increases Ca^2+^ influx per unit time and decreases the time over which efflux can occur resulting in influx becoming greater than efflux. This increases diastolic [Ca^2+^]_i_ and thence average [Ca^2+^]_i_. Via sodium-calcium exchange (NCX), this elevation of average [Ca^2+^]_i_ increases Ca^2+^ efflux to equal the influx so that influx and efflux are again equal. (2) Increased rate elevates intracellular Na^+^ concentration ([Na^+^]_i_), which decreases Ca^2+^ efflux on NCX. Therefore, a greater increase in average [Ca^2+^]_i_ is required to maintain Ca^2+^ efflux equal to influx.

There are at least 2 possible explanations for the frequency-dependent increase in diastolic [Ca^2+^]_i_ (Figure 4). One is that increasing frequency increases [Na^+^]_i_^41,97,98^ and, as discussed above, decreases NCX activity, requiring an increase in average [Ca^2+^]_i_ to maintain flux balance. This explanation is consistent with the parallel increase in [Na^+^]_i_ and diastolic [Ca^2+^]_i_.^99^ Two arguments, however, suggest that Na-independent mechanisms may also be involved. (1) The increase in diastolic [Ca^2+^]_i_ can occur abruptly on increase in rate,^94^ much faster than the presumed increase in at least global [Na^+^]_i_. (2) In the NCX knockout mouse (where changes of [Na^+^]_i_ would not be expected to increase [Ca^2+^]_i_), the effects of rate on diastolic [Ca^2+^]_i_ are similar to those in wild type.^55^ As discussed above, and in Figure 4, this Na-independent factor is likely to be the need for the increase in Ca^2+^ influx to be balanced by increased efflux and, therefore, elevated average [Ca^2+^]_i_. Why does diastolic [Ca^2+^]_i_ increase at higher rates? It might be thought that balance could occur simply by the increased frequency of Ca transients resulting in more systolic efflux. This, however, ignores that (1) the Ca transient cannot decay back to equilibrium before the next beat, resulting in end-diastolic [Ca^2+^]_i_ rising and (2) there is less time for NCX Ca removal from the cell per beat. Finally, in species with a negative force-frequency relationship (and humans with heart failure^100^), the systolic Ca transient decreases at higher rates presumably reducing the systolic efflux per beat. Both Na-dependent and independent mechanisms may contribute. The increase in Ca^2+^ entry at higher rates demands increased Ca^2+^ efflux for flux balance. This will require an increase in average [Ca^2+^]_i_. The elevated [Na^+^]_i_ will decrease NCX activity thereby requiring a greater increase in average [Ca^2+^]_i_.

### β-Adrenergic Stimulation

Physiologically, changes of β-adrenergic stimulation are the main cause of changes of heart rates. The effects of β-adrenergic stimulation will, therefore, be a combination of those of rate, discussed above, as well as direct effects. The latter include an increase in both Ca entry through the L-type Ca current and of SERCA activity (see above—via phosphorylation of PLN). The expected effects of these have been discussed earlier. In brief, the level of diastolic [Ca^2+^]_i_ will be determined by the net effects of β-adrenergic stimulation on Ca entry via the L-type current and on the shape and size of the Ca transient, which determine NCX removal. β-Adrenergic stimulation increases influx via *I*_Ca-L_, which is partly balanced by a larger Ca transient amplitude and, therefore, greater systolic efflux via NCX. This latter effect is curtailed, however, by the accelerated decay of the Ca transient resulting from greater SERCA activity.^101^ Accordingly, in rat ventricular myocytes, studied at constant rate, β-adrenergic stimulation increases diastolic [Ca^2+^]_i_.^94^ It should also be noted that β-adrenergic stimulation phosphorylates phospholemman, increasing Na-K pump activity, decreasing [Na^+^]_i_, and thereby increasing the driving force for NCX-mediated Ca^2+^ efflux. This will decrease the level of average [Ca^2+^]_i_ required to balance the increased Ca^2+^ influx.

The Ca transient and accompanying NCX removal could also be affected by changes in Ca^2+^ buffering arising from PKA-dependent phosphorylation of cardiac troponin I and PLN during β-adrenergic stimulation. The effect on cardiac troponin I will lower the affinity for Ca^2+^ binding and, except at high levels of [Ca^2+^]_i_, would be expected to decrease Ca^2+^ buffering and accelerate the decay of the Ca transient. Conversely, phosphorylation of PLN will increase affinity and increase buffering and should slow the decay. Experiments on mouse ventricular myocytes found that these effects were balanced such that there was no net effect on Ca^2+^ buffering.^101^ Further work is required to investigate the effects of β-adrenergic stimulation in a wider range of conditions.

## Clinical Aspects of Abnormal Diastolic Function

The previous sections have reviewed the fundamental mechanisms that regulate diastolic [Ca^2+^]_i_. Before turning to the changes of calcium cycling that occur in heart failure, it is important to set these in a clinical context.

### Diastolic Dysfunction

Ventricular filling in diastole relies on both a compliant ventricle and a pressure gradient between the left atrium and left ventricle (LV). In the early phase of diastole, active ventricular relaxation helps to generate this gradient by actively sucking blood into the ventricle via elastic recoil. This active phase is myocyte dependent and relies on the rapid decline in [Ca^2+^]_i_ at the beginning of diastole, leading to dissociation of the thick and thin filaments. In the subsequent, passive phases of diastole, the pressure gradient distends the ventricle.^102^ While this phase depends heavily on passive properties of the myocardium including wall thickness and fibrosis, it is also determined by diastolic [Ca^2+^]_i_ by setting the baseline myofilament activation and thus tension. Both active and passive processes require the heart to be sufficiently relaxed and compliant to fill with blood. During exercise, heart rate rises and diastolic interval decreases. Here, LV filling is maintained by increasing transmitral flow via an increase in pressure gradient. In the healthy heart, this gradient is generated by enhanced elastic recoil to reduce LV pressure in early diastole, without significantly changing left atrium pressure.^103^

Slowing of relaxation leads to diastolic dysfunction, and this is particularly pronounced during dynamic exercise with exercise intolerance—a frequent presenting feature of heart failure. Consequently, LV diastolic pressure increases, and filling can only be achieved by an increase in left atrium pressure, resulting in pulmonary congestion, breathlessness, and effort intolerance.^104^ More advanced stages of diastolic dysfunction display elevated filling pressures at rest.

Diastolic dysfunction is frequently observed alongside systolic dysfunction in heart failure with reduced ejection fraction (HFrEF). There are multiple mechanisms for slowed relaxation in HFrEF, including abnormalities in Ca^2+^ cycling (including reduced Ca uptake via SERCA)^105^ and changes in diastolic [Ca^2+^]_i_ (see below), increased extracellular collagen,^106^ increased myofilament crossbridge interactions due to metabolic changes independent of Ca^2+^,^107,108^ and loss of elastic recoil (due to failure of elastic compression in systole).^109^

Importantly, however, about half of patients with heart failure have diastolic dysfunction but a normal ejection fraction or heart failure with preserved ejection fraction (HFpEF; for review, see the article by Pfeffer et al^110^). The increase in chamber stiffness and slowed relaxation observed in HFpEF causes a rise in LV filling pressures, which, when sufficiently high, results in the heart failure syndrome.^111^ It is increasingly clear that HFpEF is not a condition of diastolic dysfunction alone and some impairment in systolic function is present at rest and becomes more prominent during exercise.^112^ This systolic impairment may further exacerbate diastolic dysfunction because contractile impairment modifies the restoring forces that drive early diastolic recoil.^113^ Additionally, HFpEF is associated with a constellation of comorbidities such as diabetes mellitus, obesity, hypertension, aging, and kidney disease. Consequently, HFpEF is accompanied by systemic changes, including inflammation and endothelial dysfunction, tissue fibrosis, microvascular dysfunction and ischemia, and multiorgan impairment such as renal failure and sarcopenia. These contribute both to the diastolic impairment and the overall clinical phenotype.^110^ In spite of its complexity, the inherent defects underlying diastolic dysfunction can be broadly grouped into 2 classes: (1) external to the cardiac myocyte and (2) resulting from impaired myocyte function.

### Myocyte-Independent Mechanisms

The extracellular matrix is a major determinant of myocardial stiffness, and increases in interstitial fibrosis and collagen are observed in HFpEF,^114^ as well as being part of the aging process.^115^ In addition to increasing stiffness,^116^ expansion of the extracellular matrix in HFpEF is associated with increased mortality and rates of hospitalization.^117^ It has also been proposed that elevations in LV filling pressure may result from increased extrinsic restraint on the heart,^118^ for example, in the obese phenotype of HFpEF where epicardial fat may cause mechanical compression of the heart, as well as exerting paracrine effects.^119,120^ Finally, it is worth noting that LV geometry itself may impact on diastolic function. Concentric hypertrophy is commonly observed clinically in HFpEF,^121^ particularly in patients with systemic arterial hypertension, and results from both expansion of the interstitium and myocyte hypertrophy.^106,122^ Here, an increase in wall thickness elevates stiffness and contributes to the diastolic impairment.^123,124^

### Myocyte-Dependent Mechanisms

Dysfunctional relaxation and higher passive stiffness in HFpEF is present at the level of the cardiac myocyte.^125^ Traditionally, diastolic dysfunction has been attributed to increased stiffness secondary to gross concentric hypertrophy (typically caused by hypertension),^126^ which is also present in isolated myocytes.^127^ However, a significant proportion of HFpEF patients do not have LV hypertrophy, and severity of hypertrophy does not closely correlate with diastolic dysfunction.^128^ Instead, the bulk of this increase in resting tension can be explained at the sarcomere.

The giant molecular spring titin, which spans the Z disk to M band, is a major determinant of passive tension by providing recoil in early diastole and resistance to stretch in late diastole.^129,130^ Its properties can be directly modified by phosphorylation (by protein kinases A and G, and CaMKII, which reduce tension)^129–131^ and oxidative modification via disulphide bonds^132^ and S-glutathionylation.^133^ As such, in addition to changes in its expression, posttranslational modifications in titin allow for dynamic changes in cellular and diastolic stiffness, which are implicated in the pathophysiology of HFpEF. Finally and intriguingly, the titin N2BA isoform exhibits a small [Ca^2+^]_i_-dependent increase in stiffness.^134,135^ Although this may add further importance to the role of diastolic Ca^2+^ in diastolic dysfunction, the significance of this finding in vivo has not yet been established.

At the sarcomere level, there is also evidence implicating the actin-myosin filaments in HFpEF. Relaxation of these depends on both diastolic [Ca^2+^]_i_ (see subsequent sections) and their sensitivity to Ca^2+^. Increased myofilament Ca^2+^ sensitivity secondary to hypophosphorylation of cardiac troponin I has been reported in HFpEF.^136^ Furthermore, abnormally high myofilament Ca sensitivity also contributes to the diastolic dysfunction observed in hypertrophic cardiomyopathy caused by sarcomeric gene mutations.^137,138^ Accordingly, the increase in resting tension in HFpEF myocytes has been linked with low PKG (protein kinase G) levels, which may impair relaxation by reducing phosphorylation of titin, cardiac troponin I, and PLN.^139,140^ A role for defective CaMKII phosphorylation of titin has also been proposed.^131^ In conclusion, although other factors may contribute to impaired diastolic function, it is important to consider the role of abnormalities in Ca^2+^ signaling.

## Diastolic [Ca^2+^]_i_ in Heart Failure

Does diastolic [Ca^2+^]_i_ change in heart failure? We will first consider data from animal and human studies where systolic function is also impaired before moving on to HFpEF.

### Heart Failure With Reduced Ejection Fraction

The decreased systolic Ca transient in heart failure may result in large part from a decrease in SR Ca^2+^ content caused by one or more of decreased SERCA activity, leaky RyRs, or increased NCX activity (see the article by Bers^105^ for review). As far as diastolic [Ca^2+^]_i_ is concerned, measurements on ventricular strips from patients with heart failure found increases in diastolic force and [Ca^2+^]_i_, which were most obvious at higher stimulation frequencies.^141^ Ca transients in cells isolated from patients had a smaller amplitude and also slowed decay,^42^ which would be expected to increase diastolic [Ca^2+^]_i_. A subsequent study found little elevation of diastolic [Ca^2+^]_i_ or force^96^ but pointed out that the lack of sensitivity of the Ca^2+^ indicator used may have made it hard to resolve changes of diastolic [Ca^2+^]_i_. Experiments on myocytes from patients with heart failure, using more sensitive fluorescent indicators (fluo-3 and fura-red), demonstrated an increase in diastolic [Ca^2+^]_i_ with increasing rate,^142^ but no control data were available. Increasing the rate of stimulation increased diastolic force in ventricular muscle strips from patients with heart failure but not controls.^143^ In a rabbit model of aortic insufficiency/restriction, the amplitude of the systolic Ca transient decreased to about 70% of control with no change of diastolic [Ca^2+^]_i_.^144^ It should, however, be noted that the experiments were performed at a slow rate (0.5 Hz). Another study on rabbit myocytes found that pressure and volume overload–induced heart failure increased diastolic [Ca^2+^]_i_,^145^ and, in contrast to much of the other work discussed here, this was unaffected by stimulation frequency. Finally, a study of right side heart failure (induced in rats with monocrotaline) showed a tendency to increased diastolic [Ca^2+^]_i_, particularly at elevated stimulation frequencies.^146^ Further complication is added by reports of a decrease in diastolic [Ca^2+^]_i_ in a sheep tachypacing model of heart failure, albeit studied at low stimulation rates.^147^ Interestingly, this was accompanied by a decrease in the L-type Ca current, which would be expected to decrease average [Ca^2+^]_i_, perhaps contributing to the decrease in diastolic [Ca^2+^]_i_. Unchanged diastolic [Ca^2+^]_i_ was found in ventricular myocytes from tachypaced dogs, but this also used low stimulation rates and whole-cell patch clamp.^148^ Decreased diastolic [Ca^2+^]_i_ has also been found in a ferret aortic banding model of hypertrophy, again at low stimulation rates.^149^

An important clinical situation that produces heart failure and depressed myocardial contractility is sepsis. Cecal ligation and puncture in the rat slowed the decay of the Ca transient; this was attributed to increased frequency of Ca sparks and accompanied by decreased systolic and increased diastolic [Ca^2+^]_i_.^150^ In another study on rats, using lipopolysaccharide administration, septic cardiomyopathy slowed the decay of [Ca^2+^]_i_.^151^ This was suggested to result from decreased activity of NCX and PMCA. This is surprising because these sarcolemmal transporters make only a small contribution (compared with SERCA) to the decay of the systolic Ca transient in small animals. In contrast, lipopolysaccharide administration in mice also slowed the decrease in the Ca transient, but this was associated with decreased SERCA activity due to sulphonylation.^152^ This was accompanied by a small decrease in diastolic [Ca^2+^]_i_ over the range of 1 to 6 Hz, the explanation for which is not clear.

Although it is not easy to draw conclusions from the above work on patients and animal models with HFrEF, it does appear that in the majority of studies where physiological rates have been studied, there is a frequency-dependent increase in diastolic [Ca^2+^]_i_ and force. More work is needed at physiological rates to characterize this.

### Heart Failure With Preserved Ejection Fraction

A major issue with studying HFpEF in animals has been the difficulty of producing an appropriate model.^153,154^ Work in 2 articles has developed potential models of HFpEF by banding the aorta in rats. In one, ventricular myocytes displayed an increase in both diastolic [Ca^2+^]_i_ and the amplitude of the Ca transient. These effects were attributed, at least in part, to increased Ca^2+^ leak through the RyR (seen as increased Ca spark frequency) and decreased NCX activity.^155^ In the other, although the animals and isolated ventricular trabeculae had impaired diastolic function, isolated myocytes, taken from the same hearts, showed lower diastolic [Ca^2+^]_i_ and shortening, suggesting that the main cause of mechanical dysfunction involved passive mechanisms rather than Ca^2+^ handling.^156^ Modeling studies have pointed out that the maintained ejection fraction in HFpEF could be achieved despite a decrease in systolic [Ca^2+^]_i_ due to the compensatory effect of concentric ventricular hypertrophy.^157^ Another, recently developed, model of HFpEF is that of an inbred rat with a hypertrophic heart. This has increased diastolic and systolic [Ca^2+^]_i_ accompanied by an increase in the L-type Ca current.^127^ It is, therefore, possible that the increase in both diastolic and systolic [Ca^2+^]_i_ augments Ca^2+^ efflux to compensate for the increased influx. In the absence of measurements, however, it is impossible to exclude a contribution from effects of [Na^+^]_i_ mediated via NCX. Interestingly, the rate of decay of the Ca transient was accelerated arguing against decreased SERCA activity.

Kidney disease is a risk factor for HFpEF, and this has been modeled experimentally by removing 80% of renal tissue resulting in prolonged ventricular relaxation and elevated end-diastolic pressure. Early work found elevated diastolic [Ca^2+^]_i_, attributed to altered NCX possibly due to increased [Na^+^]_i_.^158^ A subsequent study^159^ showed a slowing of the rate of decay of both cell shortening and the systolic Ca transient but no effect on the level of either systolic or diastolic [Ca^2+^]_i_. The experiments were, however, performed at a slow rate. This study also found that acute administration of the NCX inhibitor SEA0400 accelerated the decay of the systolic Ca transient, but the mechanism was unclear. Another study from this group found that in vivo administration of another NCX inhibitor (ORM-11035) also accelerated relaxation^160^—a result consistent with studies on the Dahl salt-sensitive rat where the improved relaxation produced by NCX inhibition was attributed to an effect on fibroblasts, decreasing fibrosis.^161^

As mentioned above, diastolic dysfunction is clinically observed in diabetes mellitus. Work on a streptozotocin rat model, with normal systolic and impaired diastolic function, found a decrease in the rate constant of decay of the systolic Ca transient due to decreased SERCA activity.^162^ Another study using the same model observed a slowing of decay, but this was accompanied by a fall not only of systolic but also diastolic [Ca^2+^]_i_ during stimulation at 1 Hz.^163^ It is unclear why diastolic [Ca^2+^]_i_ should decrease. Some studies have found decreased L-type Ca current,^164^ which may decrease average [Ca^2+^]_i_. In addition, the slowing of decay of the Ca transient will increase average [Ca^2+^]_i_ thereby allowing a lower diastolic [Ca^2+^]_i_ as long as there is sufficient time for [Ca^2+^]_i_ to fall in diastole. A similar study found a decrease in resting [Ca^2+^]_i_ in unstimulated cells.^165^ This argues for alterations of background Ca^2+^ influx or NCX/PMCA. It should, however, be noted that there is evidence that the depression of contractility in the streptozotocin rat may be independent of changes of [Ca^2+^]_i_.^166^ Metabolic dysregulation is also associated with development of HFpEF. A recent study found that the ZSF-1 obese rat had elevated diastolic but similar systolic [Ca^2+^]_i_ compared with controls.^167^ Mitochondrial [Ca^2+^] was also elevated and suggested to partly compensate by stimulating metabolism but also to result in adverse consequences of mitochondrial overload. This article also reported a decrease in diastolic [Ca^2+^]_i_ with increasing stimulation rate—a result that differs from other studies reviewed here. Atria from the ZSF-1 obese rat also show impaired function but no effect on either the rate constant of decay of the Ca transient or diastolic [Ca^2+^]_i_ was observed.^168^ Finally, a recent publication has introduced a mouse model of HFpEF using a combination of high-fat diet and nitrosative stress, which seems to mimic many of the features of the human condition.^169^ It would be useful to use this model to study [Ca^2+^]_i_.

Work on ventricular strips from patients with cardiac hypertrophy but normal ejection fraction has found interesting results. When the stimulation rate was increased, preparations from hearts showing LV hypertrophy developed increased diastolic force, and this was abolished by the contractile uncoupler butanedione monoxime suggesting that it resulted from myofilament activation. When SR function was inhibited by the SERCA inhibitor cyclopiazonic acid plus ryanodine, there was an elevation of diastolic force, which was much greater in those preparations that had previously developed significant diastolic force with raised frequency.^170^

Finally, hypertrophic cardiomyopathy can also result in a heart failure syndrome. While this is a separate disease entity to HFpEF, it also leads to diastolic dysfunction. Hypertrophic cardiomyopathy is often an inherited condition that can result from mutations in the sarcomeric proteins, which make up the thick and thin filaments, (reviewed in^171^). Many of these mutations increase the sensitivity of the myofilaments for [Ca^2+^]_i_. This, alone, would increase diastolic force, but, in addition, the increase in Ca^2+^ buffering slows the decay of [Ca^2+^]_i_, elevating end-diastolic [Ca^2+^]_i_,^29^ and, therefore, diastolic force/pressure.

### How Does Diastolic [Ca^2+^]_i_ Increase in Heart Failure?

Although, as discussed above, there is considerable variation between studies, the consensus appear to be that diastolic [Ca^2+^]_i_ increases in heart failure. There are at least 2 (nonexclusive) explanations for this. (1) As discussed in an earlier section, any decrease in the systolic Ca transient will decrease Ca^2+^ efflux during systole, requiring a compensatory increase in diastolic [Ca^2+^]_i_. (2) Another explanation is provided by the increase in [Na^+^]_i_ commonly observed in heart failure,^41,42,143,172^ which will decrease Ca^2+^ efflux on NCX, thereby requiring an increase in average [Ca^2+^]_i_, which may, in part, be provided by increased diastolic [Ca^2+^]_i_. Consistent with this, elevation of [Na^+^]_i_ by inhibition of the sodium pump increased diastolic force at elevated stimulation rates.^143^ Further evidence linking NCX to diastolic function came from work on ventricular strips from failing human hearts showing that the greater the expression of NCX, the better the diastolic function.^173^ The increase in diastolic force and [Ca^2+^]_i_ can also be attenuated by the drug ranolazine—a blocker of the late sodium current that decreases [Na^+^]_i_.^174^ Similarly, work on rats found that ranolazine reversed the diastolic impairment produced by the anticancer drug doxyrubicin.^175^ In canine myocytes, experimental ischemic heart failure increased diastolic [Ca^2+^]_i_ at elevated rates, and this was normalized by ranolazine or tetrodotoxin.^176^ Work on mice found that overexpressing CaMKIIδ_C_ decreased systolic and increased diastolic force.^35^ The decrease of systolic [Ca^2+^]_i_ has been attributed to excessive phosphorylation of RyRs leading to diastolic Ca^2+^ leak^177^ as evidenced by increased Ca^2+^ spark frequency. Again, these effects were reversed by ranolazine thereby linking them to changes of [Na^+^]_i_.^178^ In contrast, a recent study found that ranolazine had no effect on diastolic force in ventricular muscle taken from patients with HFpEF, arguing against a role for changes of [Na^+^]_i_.^179^

As mentioned in an earlier section, a different explanation of elevated diastolic [Ca^2+^]_i_ has been suggested in the cardiomyopathy observed in the mdx mouse—a model of Duchenne muscular dystrophy. Here, the elevated diastolic [Ca^2+^]_i_ is normalized by Gd^3+^ suggesting that it originates from Ca^2+^ entry through TRP channels.^77^

### Differences of Ca^2+^ Handling in HFrEF and HFpEF: a Role for NCX and [Na^+^]_i_?

**Figure 5.**
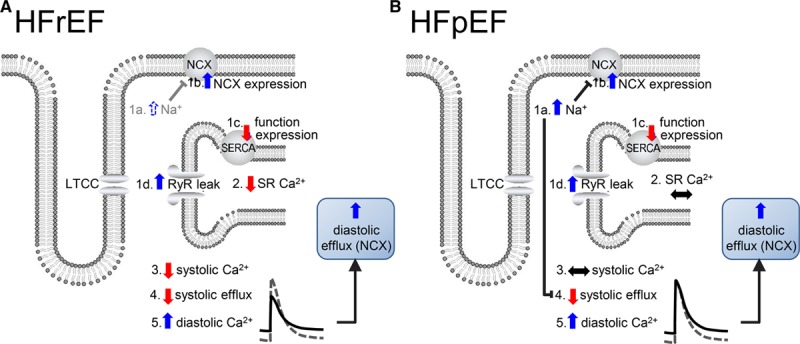
**Speculative hypothesis to account for the difference in Ca2+ handling between heart failure with reduced ejection fraction (HFrEF) and heart failure with preserved ejection fraction (HFpEF).** **A**, HFrEF. **B**, HFpEF. In both panels, heart failure results in an increase in intracellular Na^+^ concentration ([Na^+^]_i_), 1a; increase in sodium-calcium exchange (NCX) expression, 1b; decrease in sarcoplasmic reticulum Ca-ATPase (SERCA) function or expression, 1c; increase in Ca^2+^ leak through ryanodine receptor (RyR), 1d. We assume that in **A** (HFrEF) 1b–1d dominate over 1a with the net result being a decrease of sarcoplasmic reticulum (SR) Ca^2+^ content (2) and systolic intracellular Ca^2+^ concentration ([Ca^2+^]_I_; 3), systolic efflux (4) and a consequent increase in diastolic [Ca^2+^]_i_ (5), which (via NCX) raises diastolic efflux. In **B** (HFpEF), the increase in [Na^+^]_i_ (1a) dominates over 1b-d so that SR Ca^2+^ content (2) and systolic [Ca^2+^]_i_ (3) are unchanged. The increase in [Na^+^]_i_ decreases NCX activity so that diastolic [Ca^2+^]_i_ (5) has to increase to maintain systolic efflux (4) and flux balance. LTCC indicates L-type Ca channel.

An unresolved question concerns the cellular mechanisms responsible for the difference in systolic function between HFrEF and HFpEF. Figure 5 shows a speculative hypothesis. For the sake of argument, we will assume that it results from differences in Ca^2+^ signaling and that, in both cases, there is a combination of increased NCX, leaky RyR, and decreased SERCA activity resulting in decreased SR Ca content and thence the amplitude of the systolic Ca transient and systolic function. The decreased systolic efflux will require an increase in diastolic efflux so increasing diastolic [Ca^2+^]_i_. These changes could, therefore, account for HFrEF (Figure 5A). The rise of [Na^+^]_i_ often seen in heart failure will slow NCX and, if sufficient, will overcome the effects of the other changes thereby maintaining SR Ca content and systolic [Ca^2+^]_i_ at control levels. Diastolic [Ca^2+^]_i_ will be increased to maintain Ca^2+^ efflux despite the inhibited NCX. The combination would, therefore, produce an HFpEF phenotype (Figure 5B). It is, therefore, possible that the changes of Ca^2+^ cycling that underlie HFrEF and HFpEF are qualitatively identical but that, in HFpEF, the increase in [Na^+^]_i_ dominates over the other changes. Clearly, experimental studies are required to see whether this simplistic hypothesis has any validity.

## Conclusions

Control of diastolic calcium concentration is essential for normal cardiac function. As we have discussed, this regulation depends on precise balance between influx and efflux. However, there are still major uncertainties about how this is achieved. In particular, more work is required to investigate the role of the PMCA, as well as the nature of the background Ca^2+^ influx. It is essential that studies are performed at physiological heart rates. It is also important to characterize the alterations of Ca^2+^ signaling that occur in heart failure and how they may differ in failure with preserved compared with reduced ejection fraction.

## Sources of Funding

This work is supported by grants from the British Heart Foundation (CH/2000004/12801 to D.A. Eisner; FS/12/57/29717 to A.W. Trafford). D.C. Hutchings is an academic clinical lecturer supported by the National Institute of Health Research.

## Disclosures

None.
